# Directional Fluidity of Dense Emulsion Activated by Transverse Wedge-Shaped Microroughness

**DOI:** 10.3390/mi16030335

**Published:** 2025-03-14

**Authors:** Giacomo Guastella, Daniele Filippi, Davide Ferraro, Giampaolo Mistura, Matteo Pierno

**Affiliations:** Dipartimento di Fisica e Astronomia ‘G. Galilei’, Via Marzolo n. 8, 35131 Padova, PD, Italy; giacomo.guastella@phd.unipd.it (G.G.);

**Keywords:** directional roughness, maskless photolithography, microfluidic channel, fluidization, emulsion

## Abstract

The handling and fluidization of amorphous soft solids, such as emulsions, foams, or gels, is crucial in many technological processes. This is generally achieved by applying mechanical stress that overcomes a critical threshold, known as yield stress, below which these systems behave as elastic solids. However, the interaction with the walls can facilitate the transition from solid to fluid by activating rearrangements of the fluid constituents close to the wall, resulting in increased fluidity of the system up to distances greater than the spatial scale of the rearrangements. We address the impact of wedge-shaped microroughness on activating the fluidization of emulsion droplets in pressure-driven flow through microfluidic channels. We realize the micro wedges by maskless photolithography to texture one wall of the channel and measure the velocity profiles for flow directed accordingly and against the increasing ramp of the wedge-shaped grooves. We report the enhancement of the emulsion flow in the direction of the climbing ramp of the wedge activated by increasing the magnitude of the pressure gradient. A gain for the volumetric flow rate is registered with respect to the opposite direction as being to 30%, depending on the pressure drop.

## 1. Introduction

The flow of dense emulsions and pastes is essential for their processing and technological use [1,2,3,4,5]. They behave as elastic solids at low stresses and flow only when subjected to stresses exceeding a critical value, known as yield stress Their ability to flow depends on many factors, including rheology [6,7,8,9], the confinement within the capillary or microfluidic channels [10], and the interaction with the walls of the channels [11,12,13,14,15,16,17]. While the roughness of the channel has been extensively considered to increase the fluidity of dense emulsions, directional effects introduced by specific geometries are still poorly explored. It has been shown that the introduction of herringbone-shaped microroughness in pressure-driven flow introduces an activation of emulsion flow in the form of the banding of the flow in specific regions of the channel cross section, the activation being at a maximum on the tip of the herringbone grooves, when the emulsion flows in the direction of grooves narrowing [18]. Recently, 2D numerical simulations on triangular-shaped roughness reported similar activation when the flow is directed to the upward slope of the wedge-shaped obstacles, compared to the direction oriented against the vertical wall of the wedge [19]. However, no experimental confirmation has been presented so far for this wedge-like geometry.

This work aims to fill this gap by studying the velocity profiles and stress distribution of dense emulsions within a microfluidic channel, the bottom of which is textured by a periodic arrangement of wedge-shaped microgrooves, aligned perpendicularly to the flow direction. To address the role of increasing or decreasing wedges, the flow direction is alternatively inverted.

In standard (mask-based) photolithography, fabrication of gradient structures, such as modular wedges or pyramids, requires multiple masks and iterations, which is challenging, expensive, and time consuming [20,21,22].

In the last decade, maskless photolithography has progressively been established based on the direct projection of light onto the photoresistor using a computer-controlled Spatial Light Modulator (SLM) in place of the traditional, high-resolution photomask [23]. Grayscale photolithography has been widely adopted in the manufacturing of microelectromechanical systems (MEMSs), microlens arrays, Fresnel lenses, and molds for soft lithography [24,25,26]. Historically, grayscale photolithography required complex sets of physical hard masks that were applied sequentially to expose specific regions to different light levels. However, this approach had several limitations, including low spatial resolution, high costs, long fabrication times, poor process control, and a lack of flexibility, all of which hindered its broader adoption [27,28]. In contrast, digital designs allow for the precise control of light exposure according to the desired dose and target regions. This method enables faster prototyping, easy design modification, and precise control over grayscale variations to produce gradients. A digital mask allows continuous redesign and reexposure, while grayscale modulation enables varying doses for smooth transitions in structure height. This innovative technique is highly precise, versatile, and efficient, offering superior control and smoother microstructures, making it ideal for advanced microfabrication tasks [29,30,31,32]. Compared to traditional exposure through photomasks, maskless photolithography significantly simplifies and accelerates the fabrication of structures with height gradients.

The manuscript is organized as follows. In Section 2, we describe the procedure to realize the wedge-shaped microroughness by using maskless photolithography, preparing the emulsions, and controlling the emulsion flow. In Section 3, we address the fluid dynamic behavior of the textured channel by reporting the velocity profiles of the emulsion flow at different pressure loads in opposite directions with respect to the wedge roughness. Moreover, we compare the velocity profiles and the volumetric flow rates in textured and flat channels. In Section 4, we rationalize the results and review some relevant perspectives. In Appendix A, we show a comprehensive characterization of the realized structures, while Appendix B describes the experimental setup used to measure the velocity profiles.

## 2. Materials and Methods

**Figure 1:**
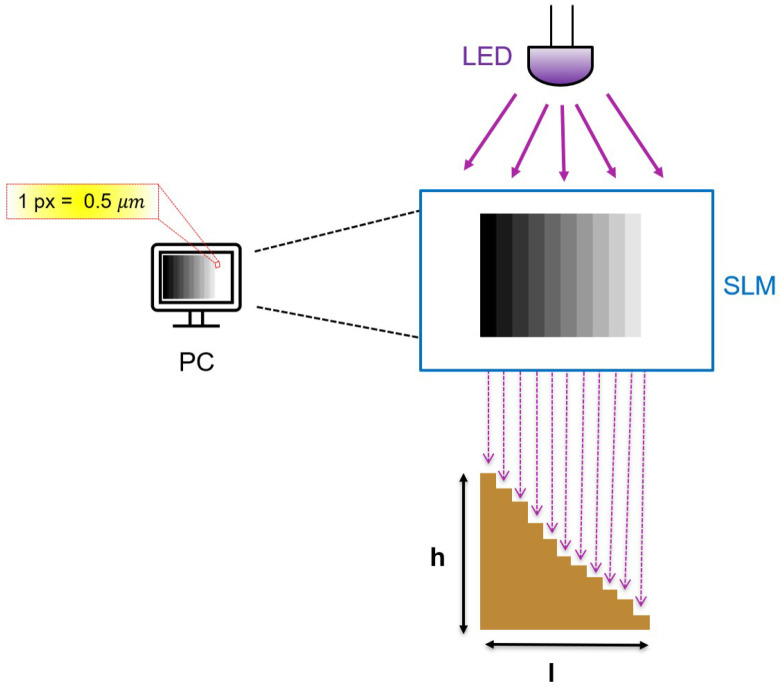
(Color online). Working principle of the μ-MLA: The digital mask is created on the PC, where each pixel corresponds to 0.5 μm. The SLM controls the intensity and phase of light to reproduce it. With the grayscale design, brighter gray levels indicate higher applied power, resulting in greater material removal.

*Microfabrication*. The microfluidic textures are fabricated using maskless photolithography with positive liquid MAP-1275G photoresist (Micro Resist Technology GmbH, Berlin, Germany) spun on a 75 × 25 mm^2^ glass slide [33]. The photolithographic setup is the Tabletop Micro Maskless Aligner μ-MLA (Heidelberg Instrument, Heidelberg, Germany), which uses a 365 nm LED of 6 W power to engrave programmable images on the photoresistor through a Spatial Light Modulator [34,35]. The mask is a digital drawing that can be easily redesigned and reexposed as needed. In addition, the direct-write process allows the laser to be modulated according to grayscale variations, as shown in Figure 1. Each grayscale value in the mask corresponds to a different dose, enabling the automatic representation of gradients [36].

Starting from the spin-coating process, 1 mL of the photoresistor is deposited on the substrate, which is spun at 3000 rpm for a uniform layer thickness $z_{\mathrm{thick}}$ of approximately 7.5 μm. Subsequently, the substrate is baked at 95 °C for 1 min to harden the resistor and improve adhesion. The selected digital mask is exposed to a dose of 180 mJ/cm^2^ using μ-MLA. The process is automatically repeated to cover an area 1 cm in width and 5 cm in length. The controlled roughness is applied exclusively to one wall of the channel, which is textured with wedges that span the entire channel width and are evenly distributed along its length. The specifications for the microfabrication process of wedges and their characteristics can be found in Appendix A. Finally, the exposed substrate is developed for 90 s within the developer solution ma-D 532/S (Micro Resist Technology GmbH, Germany).

The channel was then assembled using as the top wall a microscope slide previously drilled to apply the tubes for inlets and outlets. To close the channel, an H = 120 μm thick dry photoresist film (WBR-2000, DuPont, Wilmington, DE, USA) was used, specifically leveraging its excellent adhesion properties to surfaces [37,38]. The dry photoresist acts both as a glue to bond the top and bottom glass slides and as a sidewall of constant height. The dry photoresist was cut into a channel shape using a knife plotter (Craft Robo CC200-20, Graphtec, Yokohama, Japan) and then laminated to the glass plate, removing trapped air bubbles. To complete the bond procedure, the sample was placed under a hydraulic press for 5 min at 80 °C with a pressure of 2 bar. With this step, the cover plate was pre-bonded to the cover glass. Finally, to fix the different layers and polymerize the photoresist, the sample was exposed to UV light (i-line 365 nm) for 60 s. We characterized these microstructures using a Tencor P-17 Stylus Profiler from KLA Corporation (Milpitas, CA, USA).

The sequence of wedge-shaped textures is arranged perpendicularly to the *x* direction of the flow and has heights of 4.5 μm, somewhat similar to the mean droplet size of the emulsion. A hydrophilic coating was applied to the inner walls of the microfluidic channel to homogenize the wetting properties of the surfaces and to avoid the adhesion and coalescence of oil droplets on the walls. The coating was achieved by treating the closed microfluidic chip with an oxygen plasma to activate the inner surfaces for 1 min at 300 W and an oxygen flow of 35 sccm ($\mathrm{atm},{\mathrm{cm}}^{3}/\min$). The plasma treatment was performed using the ‘Femto’ plasma system from Diener Electronic, GmbH, Germany. Then, a 2.5% (*w*/*w*) PVP (Polyvinylpyrrolidone K90, AppliChem, GmbH, Darmstadt, Germany) water solution flowed within the channel for at least 2 h with a flow rate of 1.5 mL/h using a syringe pump [39] After treatment, the channel was rinsed by flushing 5 mL of MilliQ water (Millipore Sigma, Burlington, MA, USA) at a flow rate of 2.5 mL/h to remove excess PVP and dried with nitrogen flow at a pressure of 1 bar.

*Emulsion preparation*. The concentrated emulsion is prepared by dispersing silicone oil (polydimethyl siloxane, 1000 mPas) in a glycerine/water mixture (54% *w*/*w*) stabilized with 1% *w*/*w* tetradecyl trimethylammonium bromide (TTAB). The surfactant concentration is set to be high enough to prevent droplet coalescence but low enough to avoid flocculation by depletion [10]. The continuous phase also contains rhodamine B-labeled dye microparticles of 0.2 μm (FluoSpheres, ThermoFisher, Waltham, MA, USA), which act as fluorescent tracers. The volume fraction occupied by the oil phase relative to the total volume of the emulsion is set to Φ=0.76. This value ensures that the emulsion is stable for months and unyielding so that the roughness-induced fluidization can be addressed [16,18,40]. At the same time, it is low enough not to require a high stress, which may cause losses in the microfluidic channel.

The emulsion is prepared using a custom power homogenizer consisting of a commercial drill operating at a stabilized 24 V DC voltage and specific blades adapted to a commercial mortar (model Z247472, Merck Group, Darmstadt, Germany), which were designed and 3D-printed for this purpose. The continuous phase is placed in the mortar, and the oil phase is added at a rate of approximately 100 μL/min using a syringe pump (11 Elite Module Pico Single, Harvard Apparatus, Holliston, MA, USA). The mixture is subjected to constant and intense shearing until the oil volume fraction reaches the desired value. The voltage applied is 11.2 ± 0.1 V, corresponding to a mixing rate of 30 Hz.

**Figure 2:**
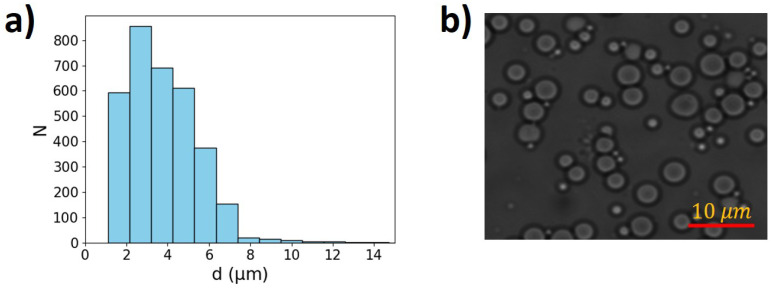
(Color online). (**a**) Droplet distribution histogram. (**b**) Image of a semi-diluted emulsion.

To measure the droplet size distribution, the emulsion was diluted in an aqueous solution containing 1% TTAB, which helps to maintain the silicone oil in the dispersed phase. A few drops of this solution were placed between two microscope coverslips. The images were then captured using a bright-field optical microscope with a 100× magnification objective, as shown in Figure 2. The mean particle diameter of the emulsion is 〈d〉 = 4 μm, with a coefficient of variation (CV) of 33.5%, derived from a standard deviation (σ) of 1.34 μm. The polydispersity index (PDI) is 1.5.

**Figure 3:**
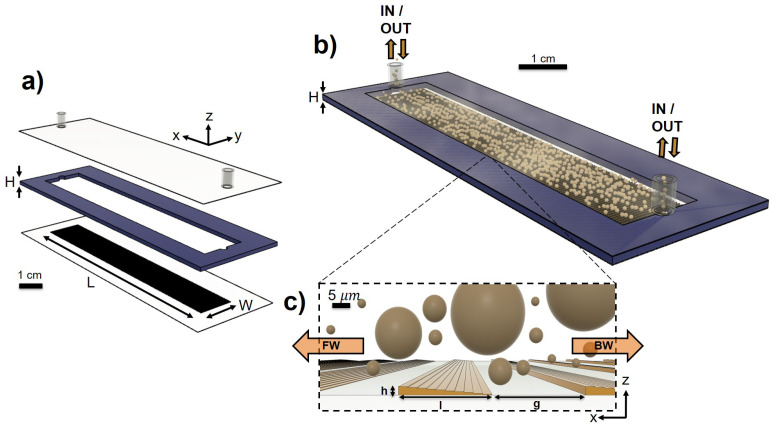
(Color online). Scale drawings of the microfluidic channel. (**a**) Exploded view, showing, from the top, the flat wall with inlet and outlet; dry photoresist with thickness *H* = 120 μm; rough wall, textured over an area of L=5 cm and W=1 cm. (**b**) Three-dimensional image of the microfluidic channel filled with the emulsion droplets. (**c**) Zoom of the side view (x,z): wedge-shaped grooves are arranged perpendicularly to the flow directions, labeled *FW* (forward) or *BW* (backward) if droplets travel against the tilted or straight side of the wedges, respectively.

*Flow Control*. The emulsion flow was pressure-controlled using a Microfluidic Flow Control System (MFCS series, Fluigent, Paris, France). Stable pressure loads in the range of (1000–7000) ± 5 mbar were applied between the inlet and the outlet of the microfluidic channel. The pressure difference was alternated to reverse the flow direction along the inclined side of the wedge-shaped grooves or in the opposite direction, referred to as *forward* and *backward*, respectively (see Figure 3a,b).

**Figure A2:**
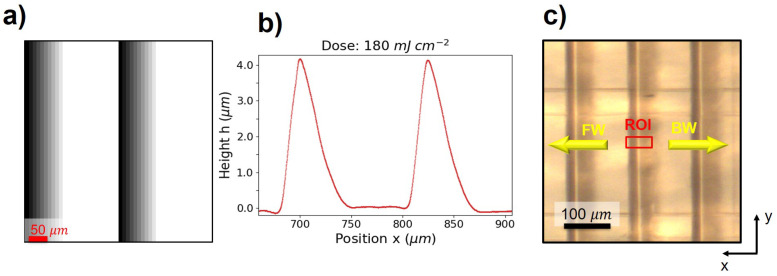
(Color online). (**a**) A digital mask was generated to create the triangular grooves’ shape. (**b**) Representative height profile shown for the pattern: *h* = 4.2 μm, *l* = 50 μm. (**c**) Top view of the wedge pattern. The flow directions are shown by the yellow arrows. The red box is the region of interest (ROI) where the flow is measured.

*Velocimetry*. The velocity profiles of the emulsion flow were measured by tracking fluorescent microtracers within a 36 × 14 μm^2^ region of interest (see Figure A2c) [40,41]. The microtracers were illuminated using a 532 nm diode-pumped solid-state (DPSS) laser (Roithner Lasertechnik GmbH, Vienna, Austria) and imaged through an inverted motorized microscope (Eclipse Ti-E, Nikon, Tokyo, Japan) in an epifluorescence configuration. The detection system consisted of a scientific CMOS camera (ORCA Fusion, Hamamatsu Photonics, Hamamatsu, Japan). A scheme of the entire experimental setup is provided in Appendix B. Images were acquired as *z*-stacks with 6 μm steps, and only trajectories crossing the optical field within the depth of focus were considered. The velocity v(z) was determined by averaging tracer displacements at the channel center. Data analysis was performed using a custom routine based on the TrackMate 7 algorithm [42,43,44].

*Geometry of the experiment*. The grooves are designed so that the height *h* of the wedges is comparable to the mean droplet size 〈d〉, while their length *l* and the groove separation *g* are about 10 times greater, with l≃g. All the main dimensions of the channel are summarized in Table 1. In particular, l/〈d〉=12.5 and h/〈d〉=1.3. This configuration results in a tiny directional parameter, characterized by a smooth tilt of the wedges, with trains of several droplets free to travel over the flat wall before encountering a wedge-shaped groove (see Figure 3b).

## 3. Results

**Figure 4:**
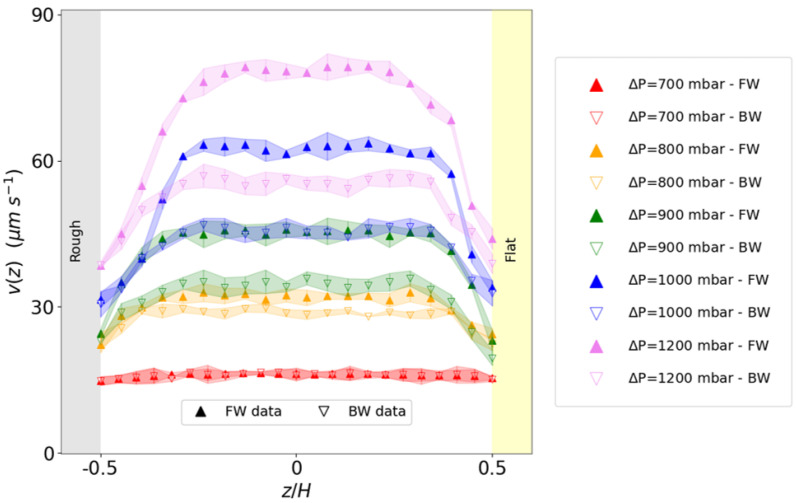
(Color online). Velocity profiles v(z) measured for different pressure drops. The distance from the channel walls is in units of channel height *H* = 120 μm. Filled upside (open downside) triangles refer to the forward (backward) direction.

In Figure 4, we report the velocity profiles of the emulsion at the volume fraction ϕ = 0.76 flowing in the patterned channel at different pressure drops. Under low-pressure drop conditions, the emulsion is flowing at constant velocity as a rigid elastic solid (plug flow) regardless of the flow direction.

**Figure 5:**
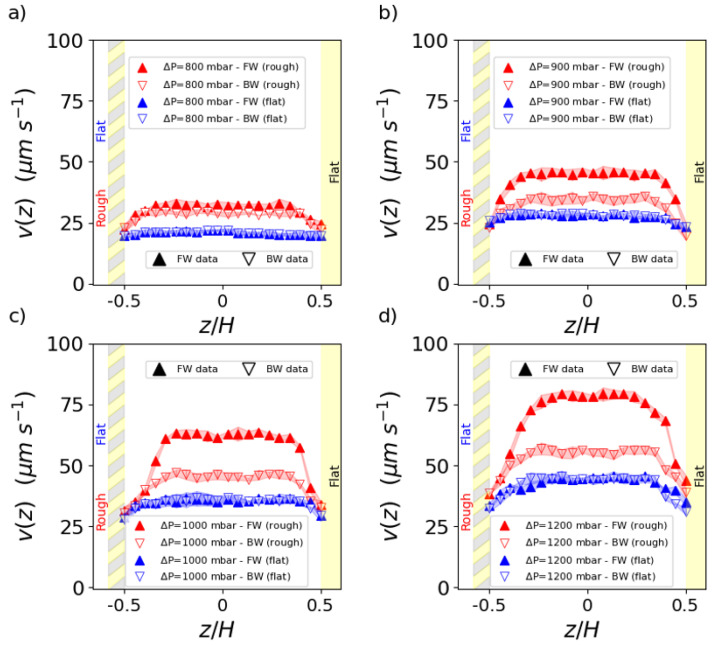
(Color online). Comparison of flow profiles in flat–flat and flat–rough surfaces under different pressure loads. Filled upside triangles and open downside triangles refer to the forward and the backward directions respectively, while red triangles refer to data acquired in a structured channel and blue to a flat one. The pressures explored are (**a**) ΔP = 800 mbar, (**b**) ΔP = 900 mbar, (**c**) ΔP = 1000 mbar and (**d**) ΔP = 1200 mbar.

**Figure 6:**
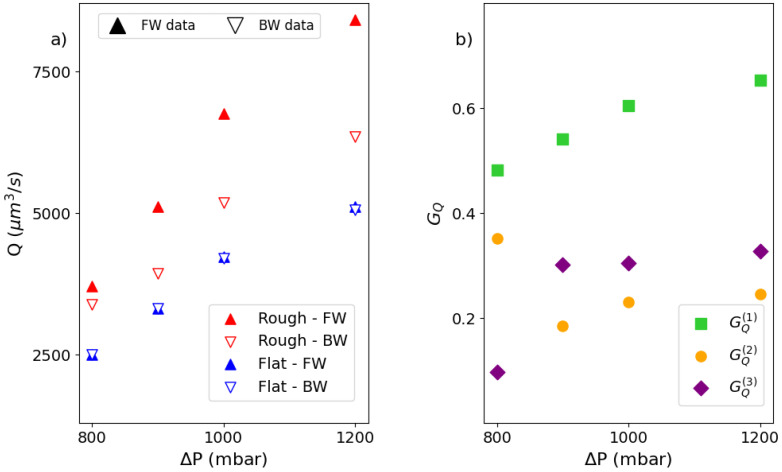
(Color online). (**a**) Comparison of the volumetric flow rate of the emulsion at ϕ=0.76 as a function of pressure load. (**b**) Comparison of the flow rate gains as defined by Equation (1).

As the pressure drop increases, the emulsion becomes progressively more fluidized, as reported by an increase in the velocity with the distance from the channel walls (shear flow). However, this increase is not the same for both forward and backward flow directions, while the magnitude of applied stress is unchanged. The effect of directionality emerges, leading to a noticeable gap between the forward and backward profiles. This gap widens as the pressure gradient increases, reflecting the growing impact of the directional roughness on the emulsion’s behavior. To evaluate the effectiveness of the wedge-shaped microroughness in enhancing the velocity profile, we systematically examined the emulsion flow in a microfluidic channel featuring two flat (unstructured) walls, at the same pressure drops applied in the textured channel. Both channels have the same height *H* = 120 μm, so that the magnitude of the pressure gradient is the same for both channels. This comparison highlights the role of directional roughness in the fluidization of the emulsion. As expected, in the flat–flat channel, the velocity profiles on forward and backward flows are the same within the experimental error bars (see Figure 5). In parallel, the presence of the wall texturing promotes more fluidization than the flat channel, regardless of the specific direction of the emulsion flow. By integrating the velocity profile over the channel cross section Σ, we obtain the volumetric flow rate $Q=\int_{\Sigma }v\left(z\right)d\Sigma$. For the textured channel, we distinguish between the flow rates $Q_{FW}$ and $Q_{BW}$, taken in the forward and backward directions, respectively. In the flat channel, the flow rate $Q_{FL}$ is obtained by averaging the velocity profiles in both directions. Clearly, the flow rate increases as the pressure drop increases. For the flat channel, it is the same within the error bars, regardless of the flow direction, as expected. In contrast, for the textured channel, the flow rate does not increase with pressure by the same amount in both directions, the increase being more consistent in the forward direction (see Figure 6a). To quantify the efficiency of the roughness design in increasing the emulsion transport at constant applied stress, we introduce the flow gain $G_{Q}$ as

(1) $$\begin{array}{cc} G_{Q}^{\left(1\right)} & =\frac{Q_{FW}-Q_{FL}}{Q_{FL}};\\ G_{Q}^{\left(2\right)} & =\frac{Q_{BW}-Q_{FL}}{Q_{FL}}\;;\\ G_{Q}^{\left(3\right)} & =\frac{Q_{FW}-Q_{BW}}{Q_{BW}}\;, \end{array}$$

With $G^{\left(1\right)},G^{\left(2\right)}$ being the flow gains of the forward (backward) flow with respect to the flat channel, respectively; $G^{\left(3\right)}$ being the flow gain of the forward flow with respect to backward, provided that all flows are compared at the same pressure load with that different pressure loads (Figure 6b).

**Figure 7:**
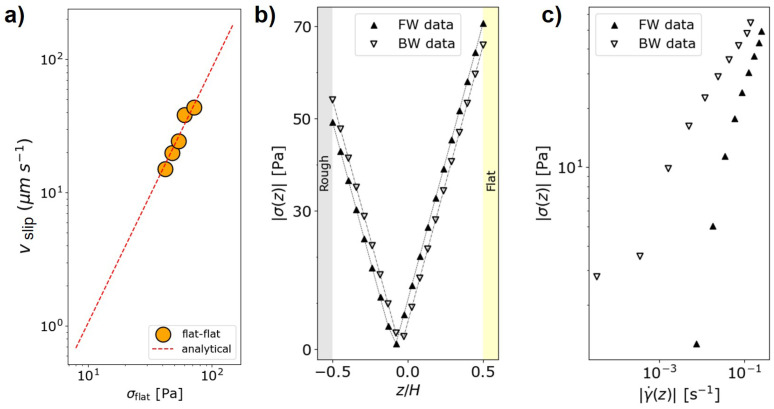
(Color online). (**a**) Slip velocity as a function of the corresponding shear stress computed at the solid wall for a flat–flat channel with the same height of the rough-flat channel. (**b**) Absolute value of the stress profile |σ(z)| within the microfluidic channel for pressure drop ΔP = 1200 mbar. (**c**) Flow curves extracted from the stress data reported in panel (**b**) as a function of shear rate $\dot{\gamma }$ computed from the derivative of the velocity profiles in Figure 5d, for the emulsion driven at ΔP = 1200 mbar.

As shown in Figure 6b, when the emulsion flows in the direction of the raising ramp (forward) of the wedge-shaped grooves, the flow rate gains about 25% compared to the opposite direction, for all pressure drops explored but the lowest. Indeed, for ΔP = 800 mbar, the emulsion is only at the beginning of the roughness fluidization process. Conversely, with respect to the flat channel, the gain in the forward direction is maximum and increases until about 60% as the pressure drop increases, while it is similar to that computed considering forward and backward directions and is rather independent of the pressure, except for the lowest one. To investigate the stress profile within the channel, we make use of a specific protocol developed and validated by numerical simulations [19]. In fact, due to the presence of different boundary conditions at the channel walls (flat and rough), the stress profile within the channel is not directly accessible by simply knowing the pressure drop and the length of the channel. The protocol is rooted in the calibration between the slip velocity, i.e., the velocity of the emulsion flow at the contact with the solid wall and the shear stress, within a channel featuring both walls flat, and the same height of the flat–rough channel. The corner stone of the protocol consists of the validation that allows assigning the stress at the flat wall in a flat–rough microfluidic channel once the relation between stress and slip velocity in a flat–flat channel is measured for the same emulsion (see Figure 7a).

The mechanical-balance condition $\partial_{Z}\sigma \left(z\right)=\nabla P$ implies a linear stress profile σ(z) as a function of the vertical position *z*, where the slope is given by the pressure gradient ∇P. The stress profiles are different for the opposite flow directions, corresponding to different sides of the wedged grooves encountered by the emulsion droplets (see Figure 7b). Notably, this implies that the rheological response of the emulsion is *directional* in the sense that it is different for the different configurations of the wedge-shaped roughness, here obtained by reversing the emulsion flow (see Figure 7c). This result is similar to that reported either for channels textured by herringbone-shaped roughness or numerical wedge-shaped roughness, although the details of the experimental wedge-grooves do not match those of the simulations [18,19]. Furthermore, this aligns with the report of a different rheological response when boundary wettability is changed and an increase in shear stress is observed for a hydrophobic wall compared to a hydrophilic one [12,15]. In this case, that is, in the presence of wedge-shaped grooves, the rheological response is not determined by a change in the wettability pattern but is due to a change in flow direction with respect to a fixed suitable physical texture of the wall roughness.

## 4. Discussion and Conclusions

From a micromechanical perspective, the roughness-induced fluidization of dense emulsion is attributed to an increase in the number of plastic rearrangements [10,16,45]. Droplets interacting with the textured wall undergo more variations in speed and direction compared to the flat wall, resulting in a scrambling effect that promotes interactions with neighboring droplets and facilitates flow. This is marked by some key changes in the velocity profiles, such as the increase in the velocity, the growth in the shear flow regions close to walls, and the corresponding thinning of the plug flow region in the center of the channel height, as reported by Figure 4 and Figure 5. The directional design has shown that the roughness geometry plays a key role. For herringbone-shaped roughness, a flow banding along the channel cross section has been reported, with flow boost at the herringbone tip [18]. For wedge-shaped roughness, numerical simulations have elucidated the lift-off of emulsion droplets climbing on the wedges as the driving mechanism that promotes directional fluidization [19]. Here, we confirm this view by showing a significant gain in the volumetric flow rate along the direction that promotes the droplets’ lift-off, compared to the opposite direction or the flat wall. The directional fluidization takes place at a high enough droplet concentration and pressure. This is consistent with the reporting within different geometries for the directional roughness [18]. In fact, at a low volume fraction, the emulsion is fluid-like for vanishing pressure drops. Therefore, it is rather insensitive to the specific wall texturing. Conversely, for dense emulsions, the pressure load should overcome a threshold to trigger fluidization and, even more, to activate directional fluidization. Indeed, plug (or almost plug) flows are independent of or less sensitive to directional details of the roughness texturing. Our results confirm that the gain in the flow rate increases with increasing pressure. In the limit of a very high pressure load, a recovery to a fluid system is expected and also reported [18]. However, in the present study, we could only address a limited range of pressure drop since ΔP=1200 mbar is the highest pressure before the channels start leaking.

Concluding, to investigate the directional fluidization of emulsions flowing through a microfluidic channel with one wall textured by wedge-shaped grooves, we successfully employed grayscale maskless photolithography to fabricate structures with a height gradient. The optimization of the exposure parameters for the photoresist enabled the fabrication of grooves with a height of approximately 4 μm, demonstrating the precision and effectiveness of this process for microfluidic applications. At the fluid-dynamic level, the wedge-shaped design promotes a directional fluidization of dense emulsion, being more effective in the direction that follows the rising side of the wedge. By running a protocol specifically validated to measure the stress profile in channels with different boundary conditions on opposite walls, we obtained the stress distribution in the channel and reported the presence of two flow curves $\sigma (\dot{\gamma })$ according to the two flow directions, forward and backward with respect to the wedge side [19]. This reflects the selective increase or decrease in the emulsion flow rate promoted by directional roughness, regardless of the specific geometry considered. Considering different combinations (l,g,h) of the wedge-shaped microgrooves, it is natural to ask which one would produce the optimal flow throughput in a microfluidic channel at a given pressure gradient. The resulting space of parameters would be very demanding for practical realization, and the help of numerical simulations would be invaluable to individuate the optimal set.

The work presented here has significant implications for several emerging processes such as bioprinting, where emulsions are used as scaffold structures to support cell growth in efforts to replicate living tissues [46,47,48]. While microfluidics enables precise control over fluid flows, the printability of various bioinks is often compromised by the increased stress during extrusion, which can damage cells and negatively affect print quality. Our findings reveal a notable improvement in fluid flow under the same pressure gradient when compared to unstructured channels. This enhancement holds promise for bioprinting applications as it enables higher fluid throughput. Consequently, this reduces the time cells are exposed to damaging forces, such as those within the nozzle. By minimizing this exposure, we can improve cell viability and ensure better material quality post-printing [49]. Furthermore, incorporating directional roughness into the printing channels could further optimize fluid flow and cell arrangement.

## Figures and Tables

**Table 1:** Main characteristic dimensions of the channel.

H (μm)	L (cm)	W (cm)	H (μm)	L (μm)	g (μm)	$\theta \;{(}^{\circ })$
120 ± 5	5.00 ± 0.01	1.00 ± 0.01	4.2 ± 0.2	51 ± 4	75 ± 1	4.7 ± 0.4

**Figure A1:**
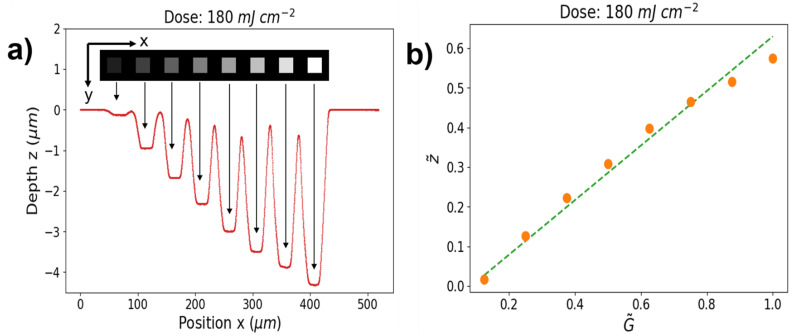
(Color online). (**a**) Dose matrix digital mask and profilometer measurements of the relative structure obtained. (**b**) Normalized and inverted calibration curve with fitted data. $\tilde{G}=G/128$ and $\tilde{z}=z/z_{\mathrm{thick}}$.

**Figure A3:**
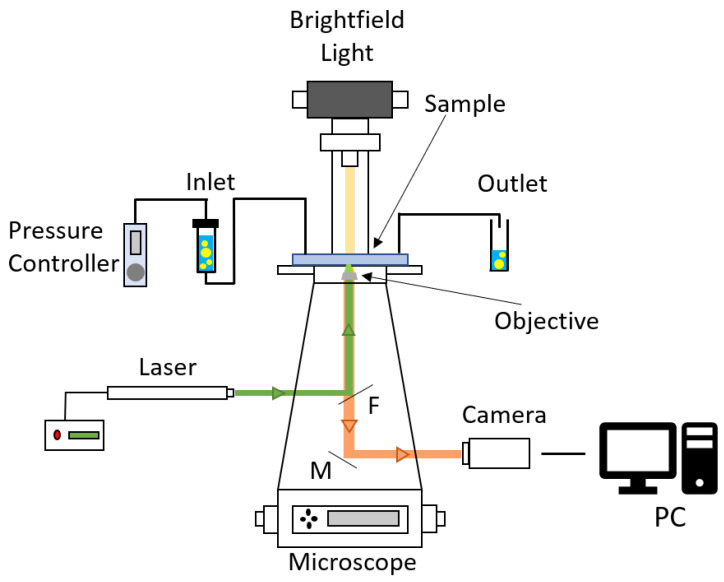
(Color online). Scheme of the experimental setup adopted for the measurements.

## Data Availability

The essential data are contained within the article. The raw data are available on request from the corresponding author.

## Abbreviations

The following abbreviations are used in this manuscript:

ROI	Region of Interest
μ-MLA	Micro Maskless Aligner
SLM	Spatial Light Modulator
FW	Forward
BW	Backward
MEMSs	Microelectromechanical Systems
CMOS	Complementary Metal-Oxide Semiconductor
TTAB	Tetradecyl Trymethyl Ammonium Bromide
PVP	Poly Vinyl Pyrrolidone
MFCS	Microfluidic Flow Control System

## Appendix A. Microfabrication

To realize 3D structures with a vertical resolution of 4 μm, the relation between gray level and photoresistor removal is needed, which is determined by calibration. The digital mask consists of eight 50 × 50 μm^2^ squares uniformly filled with a single gray level G (ranging from 1 to 128) chosen to achieve material removal depths greater than 4 μm, the target range of interest. The applied digital mask is visible in Figure A1a.

Post-lithography profilometry measurements of this geometry provide the average depth of material removed within each square (Figure A1a). To correlate height with color in the 3D design, the average points for each square are inverted, normalized at $\tilde{G}=G/128$ and $\tilde{z}=z/z_{\mathrm{thick}}$, and fitted for mapping purposes (Figure A1b).

The data obtained show a linear relationship between the depth reached and the grayscale value up to ${\tilde{z}}_{thick}=0.5$, after which the data deviate from the expected trend. This deviation can be caused by both the absorption of the photoresistor and by a bleaching effect.

Using photoresistor calibration, we obtain all the parameters for the fabrication of wedge structures with height *h* = 4 μm and length *l* = 50 μm. The specifications are $\mathrm{Dose}=180\;\mathrm{mJ}/{\mathrm{cm}}^{2}$ and development time $t_{\mathrm{dev}}=90\;s$.

First, in Figure A2a, the digital mask created is visible to obtain the desired shape. The mask is designed as a series of 10 adjacent rectangles arranged vertically, all having the same length and height. The length of these rectangles must be equal to the width of the mask, while the height is varied depending on the width of the wedge to be obtained. Each of these strips is filled with a different gray tone value, which decreases from 120 to 0 in steps of 12. The background, on the other hand, is completely white and corresponds to a full exposure of the substrate.

To assess the quality of the micro-wedges, a tip profilometer was used, as can be seen in Figure A2b.

Although depth variations were observed, the residual depth error within any analyzed region remained below 60 nm, which was negligible with respect to height (*h* = 4.2 μm). For simple structures such as stairs and wedges, our method accurately reproduced the overall shape compared to the original design.

The characteristics described above were analyzed for three samples, each consisting of 250 wedges and each belonging to a fabrication performed on different days. It should be noted that the samples related to the gaps are less numerous because the measurements were taken in pairs to avoid data correlation.

**Table A1:** Profilometer characterization value of wedges’ main characteristics.

Sample N.	$\bar{h}$ (μm)	$\bar{l}$ (μm)	$\bar{\theta }\;{(}^{\circ })$	$\bar{g}$ (μm)
1	4.2 ± 0.2	51 ± 4	4.7 ± 0.4	75 ± 1
2	3.9 ± 0.3	50 ± 5	4.9 ± 0.6	78 ± 1
3	4.3 ± 0.2	55 ± 3	4.4 ± 0.4	74 ± 1

## Appendix B. Experimental Setup for Measuring the Velocity Profiles

The emulsion flow was controlled using a Fluigent MFCS™-EZ microfluidic pressure controller. The system maintained a stable pressure differential between the channel inlet and outlet by automatically adjusting the applied pressure. Fluid delivery was managed through Teflon tubing, ensuring consistent fluidic resistance by maintaining a fixed length. Since the pressure gradient magnitude remained constant in both directions, we directly compared the emulsion flow under equivalent applied stress but with different roughness geometries. Fluorescent tracers labeled with Rhodamine B were introduced into the continuous phase for flow visualization. The experimental setup featured an inverted optical microscope with a DPSS laser for fluorescence excitation in an epi-fluorescence configuration (see Figure A3). Brightfield illumination was used for initial alignment and emulsion observation. To record the tracks of the tracers, a sCMOS camera was coupled with the motorized *z*-stage via a mirror (M), which redirected the light toward the camera. The laser illuminated the sample, and fluorescence imaging was performed using a dichroic mirror (TRITC bandpass emission filter (F), spectral bandwidth 570–620 nm) to direct excitation light onto the emulsion while collecting emitted light. A 60× apochromatic long working distance objective (WD = 2 mm, NA = 0.7, SuperPlan Fluo, Nikon, Tokyo, Japan) provided high-resolution visualization of tracer motion and emulsion dynamics within the microfluidic channel.
